# Mental health differences between German gay and bisexual men and population-based controls

**DOI:** 10.1186/s12888-017-1435-7

**Published:** 2017-07-21

**Authors:** Frank A. Sattler, Gabriele H. Franke, Hanna Christiansen

**Affiliations:** 1 Department of Clinical Child and Adolescent Psychology, Philipps University Marburg, Gutenbergstraße 18, 35032 Marburg, Germany; 2 Psychology of Rehabilitation, University of Applied Sciences, Stendal, Germany

**Keywords:** Gay and bisexual men, Population-based controls, Minority stress, Minority health, Germany

## Abstract

**Background:**

International studies have revealed that gay and bisexual men present more mental health problems than the general male population. Furthermore, there is evidence that minority stress predicts mental health problems in gay and bisexual men. The aim of the present study is to provide initial data on mental health differences in Germany and to analyze the effect of minority stress.

**Methods:**

Mental health data on *n* = 1903 German gay and bisexual men and *n* = 958 men from a population-based sample were assessed using a shortened version of the SCL-90-S. The mental health of the two samples was compared. Furthermore, a linear regression was conducted for the gay and bisexual sample: mental health was used as the criterion and minority stressors as predictors.

**Results:**

As compared to our population sample, gay and bisexual men demonstrated more mental health problems with a moderate effect size. In the regression, minority stress predicted mental health problems in the gay and bisexual sample.

**Conclusions:**

We observed pronounced mental health differences between gay and bisexual men versus the population sample. These differences could be at least partly due to the minority stress gay and bisexual men face. Research should focus on how to reduce and cope with minority stress.

**Electronic supplementary material:**

The online version of this article (doi:10.1186/s12888-017-1435-7) contains supplementary material, which is available to authorized users.

## Background

Meta-analyses including data from several countries (e.g., the Netherlands, New Zealand, and U.S.) indicate that compared to heterosexual men, gay and bisexual men have a higher prevalence of mental disorders such as depressive, anxiety, substance use, and obsessive-compulsive disorders [1–3]. Moreover, preliminary data suggests that gay and bisexual men are at an increased risk for attention-deficit/hyperactivity disorder (ADHD), posttraumatic stress disorders (PTSD), eating and psychotic disorders [4–6].

Meyer’s minority stress theory [2, 7] claims that the relationship between sexual orientation and mental health is mediated by minority stressors that include prejudice events, rejection expectations (also called rejection sensitivity) [8], sexual orientation concealment, and internalized homophobia (also called internalized homonegativity) [9]. These variables are similar to a conceptualization proposed by Herek, Gillis, and Cogan [10]. While prejudice events are an aspect of enacted stigma (overt behavioral expression of sexual stigma), rejection sensitivity and sexual orientation concealment are part of felt stigma (knowledge of society’s stance toward sexual minorities including expectations about the likelihood of stigma being enacted in a given situation), and internalized homonegativity is a form of internalized stigma (the personal acceptance of sexual stigma as part of one’s own value system or self-concept).

In several studies targeting primarily North American and European gay and bisexual men and other sexual minority individuals, discrimination events, rejection sensitivity, and internalized homonegativity have consistently been found to predict mental health problems [5, 11–16]. On the other hand, findings on concealment have been ambiguous. While concealment was associated with worse mental health in some studies targeting U.S. gay and bisexual men [17, 18], it was also associated with fewer mental health problems in gay and bisexual men in a Californian population-based sample [19]. Also, disclosure (usually considered as the opposite of concealment) predicted more mental health problems in German gay men when controlling for certain variables (other minority stressors, coping, and social support), while this prediction was at an insufficient level of *β* < .1 [16]. The association between concealment and fewer mental health problems may be linked to findings that indicate greater victimization experienced among disclosed lesbians, gay, and bisexual individuals (LGB) [20, 21] and a strong correlation between disclosure and internalized negativity (*r* = .48) [16]. Due to these inconsistencies on how concealment modulates mental health problems, we decided to exclude this variable from the present study.

Despite the large number of international studies comparing the mental health of gay and bisexual men versus heterosexual men [1–3], there are no studies using German samples. The aim of this study was thus to close this gap by comparing the mental health of German gay and bisexual men with that of a population-based male sample. The hypotheses tested in this study were: hypothesis 1) gay and bisexual men will report more mental health symptoms than a male population sample, and hypothesis 2) minority stress (victimization, rejection sensitivity, and internalized homonegativity) will predict mental health problems in gay and bisexual men.

## Method

Our gay and bisexual sample and population-based male sample were recruited independently. There were therefore some differences in the recruiting plan and questionnaires used in the two samples. The authors did not decide to combine the aforementioned data for the present study until after the recruitment of both groups.

### Gay and bisexual participants

German gay and bisexual men were recruited online in 2014 via mailing lists of Philipps University Marburg (PUM), sexual minority associations, news portals, and sexual minority social media. The recruitment was part of the project Minority Stress, Coping, Social Support, and Mental Health in German Gay and Bisexual Men (MHGGB). The participants read an informative text about the study and provided written informed consent online. Then they were directed to access the online questionnaire. Anonymity was ensured by our not collecting any personal data (such as name, address, or IP address). Also, all staff members who had access to the data were bound to confidentiality. All data was saved and processed on a German server.

A total of *n* = 1903 individuals participated in the study. Inclusion criteria were a minimum age of 18 years, identifying as male, identifying as gay or bisexual, and fluency in German. The following participants were excluded from our analyses: *n* = 472 did not complete the questionnaire and *n* = 7 were under 18 years of age or did not report a valid age.

After the exclusions, our final sample consisted of *N* = 1424 self-identified gay and bisexual men with a mean age of 38.0 years (*SD* = 11.4, *range* = 18 to 77 years). Ethnicity/nationality was as follows: 89.7% (*n* = 1277) were autochthonous Germans and 10.3% (*n* = 147) were immigrants to Germany or had at least one immigrant parent. 48.3% (*n* = 688) had a male partner, 3.5% (*n* = 50) had a female partner (of those *n* = 6 had a male and a female partner), and 48.6% (*n* = 692) were single. Educational levels were as follows: .2% (*n* = 3) had no school degree, 4.0% (*n* = 57) had a junior high school degree, 13.6% (*n* = 193) had a middle high school degree, 29.6% (*n* = 421) had a senior high school degree, and 52.6% (*n* = 750) had a university degree.

### Population sample of male controls

Our population sample consisted of *N* = 958 German men recruited as the population-based norm group for the German SCL-90-S in 2014. The process of recruitment is explained in detail elsewhere [22]. The sample’s mean age was 46.8 years (*SD* = 15.6, *range* = 18 to 75). The population sample consisted of 97.2% (*n* = 931) German nationals, 2.4% (*n* = 23) with another nationality, and 0.4% (*n* = 4) who provided no nationality. Partnership status was as follows: 67.4% (*n* = 646) had a partner, 29.2% (*n* = 280) were single, and 3.4% (*n* = 32) did not provide an answer. The participants in the population sample did not indicate their partner’s gender. Their educational levels were as follows: 4.8% (*n* = 46) had no school degree, 36.6% (*n* = 351) had a junior high school degree, 26.0% (*n* = 249) had a middle high school degree, 14.3% (*n* = 137) had a senior high school degree, 17.8% (*n* = 171) had a university degree, and .3% (*n* = 4) did not provide information on their educational level.

The population-based sample did not state their sexual orientation [22]. However, we were interested in how many gay and bisexual men were probably included in this sample. Since no study has been published to date on the prevalence of a gay, bisexual, and heterosexual identity in the German population, we had to base this estimate on U.S. findings: as about 2.8% of men in the U.S. identify as gay or bisexual [23], approximately *n* = 26 participants in our population sample should identify as gay or bisexual. We therefore assume that our population of male controls mainly consists of heterosexuals.

### Measures

Since the project MHGGB used a broad number of variables (including variables on minority stress, social support, coping, and mental health) all scales used were substantially shortened in order to not overload the participants.

#### Victimization

Victimization was assessed with a five-item victimization scale (VS) in gay and bisexual men that was previously published [16]. It is known to have a one-factor structure and a sufficient Cronbach’s alpha of .72 [16] and was based on a scale by Herek and Berrill [24]. The VS consists of 5 items asking about victimization events since the age of 16 years. It uses a 5-point response scale (0 = *never* to 4 = *four times or more often*). Cronbach’s alpha from the scale in our gay and bisexual sample was satisfactory with .73.

#### Rejection sensitivity

Rejection sensitivity was assessed with a modified version of the Gay-Related Rejection Sensitivity Scale [8] in our gay and bisexual sample. The scale was previously validated and published in a study with German gay men [16]. It consists of three items assessing concern of rejection using a 5-point response scale (1 = *strongly disagree* to 5 = *strongly agree*). Its internal consistency was found to be excellent (Cronbach’s α = .89) [16]. Cronbach’s alpha was .73 in this study.

#### Internalized homonegativity

Internalized homonegativity was assessed with a previously published three-item scale [16] in our gay and bisexual sample. The three items derived from the personal homonegativity subscale of the Internalized Homonegativity Inventory [9] and were found to have a good Cronbach’s alpha of .86 [16]. In the present study, Cronbach’s alpha was .84.

#### Mental health

In both the gay and bisexual men and in the male population sample, mental health was assessed with items of the German Symptom-Checklist-90-Standard (SCL-90-S) [22], the new version of the German SCL-90-R [25]. The SCL-90-S contains 90 items assessing mental health symptoms in the last 7 days, and is composed of the nine subscales, anger-hostility, anxiety, depression, paranoid ideation, phobic anxiety, psychoticism, somatization, interpersonal sensitivity, and obsessive-compulsive symptoms. The SCL-90-S was shortened for the MHGGB project to contain three items for each of the nine mental health subscales. The item selection was examined by all authors until we agreed that face validity criteria had been met. In the case of somatization and interpersonal sensitivity, we combined two items into a single one: item 4 of the somatization subscale (“faintness or dizziness”) and item 40 (“nausea or upset stomach”) were combined to read “dizziness or nausea”, while item 9 on the obsessive-compulsive symptom subscale (“trouble remembering things”) and item 55 (“trouble concentrating”) were combined to read “trouble remembering things and/or concentrating”. For the subscales somatization and interpersonal sensitivity, two more items from the original scales were used in their original form. The items on all other subscales were used without modification.

While the gay and bisexual participants filled in this adapted version of the SCL-90-S, the population sample filled in the original SCL-90-S. We therefore found it necessary to average items 4 and 40 as well as items 9 and 55 of the population sample in order to make the scores of the gay and bisexual and population samples comparable. These derived scores were used as part of the 3-item subscales in the adapted SCL-90-S (see the Additional file 1: Table S1 for a detailed display of items used).

In order to test the factorial validity of our newly derived mental health scale, maximum-likelihood factor analyses with promax rotation (*κ* = 4) were computed for the adapted SCL-90-S scale. The analyses were conducted separately for the two samples. For the gay and bisexual sample, two factors were extracted with every item loading *λ* > .4 on the first factor and one anxiety item (“spells of terror and panic”) loading *λ =* .41 on the second factor. Since the second factor consisted only of one item that scored even higher on the first factor (*λ* = .60), we preferred a single-factor solution. For the population-based sample, a single-factor solution resulted with every item loading *λ* > .4 on this factor. These findings are in line with previous results on the SCL-90-R, indicating that the measure is best described as a unidimensional measure for psychological distress rather than as a multidimensional measure [26, 27].

Cronbach’s alpha of the adapted SCL-90-S score was .95 for our total sample (with the gay and bisexual and population samples combined), while the total score of the original SCL-90-S was .98 [22].

### Statistical analyses

All statistical analyses were conducted in IBM SPSS Statistics 22. Missing data (*n* = 4 of the population sample) were excluded from further analysis. Independent *t*-tests were conducted on differences in the sociodemographic variables age and educational level between gay and bisexual versus population-based men. Age was coded in years and educational level was coded from 1 = *no school degree/junior high school degree* to 4 = *university degree*. We combined the groups with no school degree and junior high school degree because the sub-group of gay and bisexual men without a school degree consisted of only *n* = 3 individuals.

Means and standard deviations of the mental health scale were calculated for both groups. A weighted ANCOVA was conducted to compare the mental health of gay and bisexual men with the one of the male population sample. Sociodemographics were used as covariates. Cohen’s *d* was calculated for each significant pairwise comparison in the ANCOVA.

In addition, bivariate correlations between minority stress and mental health were computed for the gay and bisexual sample. Furthermore, independent *t*-tests were conducted between gay and bisexual men on levels of minority stress and mental health. Finally, a step-wise linear regression on mental health was computed for the gay and bisexual sample using sociodemographics and minority stressors (victimization, rejection sensitivity, and internalized homonegativity) as predictors.

## Results

### Differences in sociodemographics

The levels of the two sociodemographic variables age and educational level were compared between both samples. Independent *t*-tests revealed that our gay and bisexual sample was younger, *t*(2376) = −15.83, *p* < .001, and reported a higher education level, *t*(2376) = 29.90, *p* < .001, than our population sample.

### Mental health comparisons

Our gay and bisexual sample’s mental health problems’ mean was .60 (*SD* = .60, *range* = 0 to 3.2), while the population sample’s mean was .34 (*SD* = .43, *range* = 0 to 3.0). The mean value of mental health problems measured .50 (*SD* = .55, *range* = 0 to 3.2) when both samples were combined.

An ANCOVA (weighted for group size) was computed with mental health of gay and bisexual men versus the population sample as outcomes. Age and educational level were used as covariates. The ANCOVA revealed the following results: the two groups differed significantly in mental health, *F*(1,2360) = 104.47, *p* < .001, with a medium effect size, *d* = .43. Age influenced mental health significantly, *F*(1,2360) = 36.78, *p* < .001, with a small effect size, *d* = .25: older participants displayed fewer mental health problems. In addition, the educational degree influenced mental health significantly, *F*(1,2360) = 45.31, *p* < .001, with a small effect size, *d* = .28. The better educated participants thus reported fewer mental health problems.

### Minority stress and mental health

Bivariate correlations of the constructs were computed within the gay and bisexual sample only (see Table 1). Victimization correlated positively with rejection sensitivity, *r* = .25, *p* < .001, and internalized homonegativity, *r* = .09, *p* < .01. Rejection sensitivity was associated with internalized homonegativity, *r* = .23, *p* < .001. Mental health was positively associated with all three minority stress scales, *r* = .31 to .39, *p* < .001 (Table 1).

**Table 1:** Bivariate correlations of the scales in the gay and bisexual sample

Scale	1.	2.	3.	4.
1. Victimization				
2. Rejection sensitivity	.25***			
3. Internalized homonegativity	.09**	.23***		
4. Mental health	.34***	.31***	.39***	

**p < .01

***p < .001

Comparisons between gay and bisexual men in minority stressors revealed that gay men reported more victimization, *t*(1422) = 2.46, *p* < .05, and less internalized homonegativity, *t*(1422) = −7.05, *p* < .001, than bisexual men. No differences were found in rejection sensitivity, *t*(1422) = −.11, *p* > .05, or mental health, *t*(1422) = 1.17, *p* > .05.

Furthermore, a step-wise regression analysis was computed for the gay and bisexual sample to analyze possible reasons for the mental health differences that the ANCOVA revealed. Mental health was used as the criterion. In step 1, sociodemographics were included as predictors, while victimization, rejection sensitivity, and internalized homonegativity were introduced as predictors in steps 2 to 4 (see Table 2).

**Table 2:** Step-Wise linear regression on mental health

Steps	β	R^2^
Step 1		.04***
Age	−.12***	
Education level	−.16***	
Step 2		.15***
Victimization	.34***	
Step 3		.20***
Rejection sensitivity	.23***	
Step 4		.31***
Internalized homonegativity	.35***	

Education level coded as 1 = *no school degree/junior high school degree*, 2 = *middle high school degree*, 3 = *senior high school degree*, 4 = *university degree*

***p < .001

In step 1, an older age, *β* = −.12, *p* < .001, and a higher education level, *β* = −.16, *p* < .001, predicted significantly fewer mental health problems. Model 1 thereby explained 4% of the criterion’s variance. In step 2, victimization significantly predicted mental health problems, *β* = .34, *p* < .001, and 15% of the criterion’s variance was explained. In step 3, rejection sensitivity significantly predicted mental health problems, *β* = .23, *p* < .001, with 20% of the variance in mental health problems explained. In step 4, internalized homonegativity significantly predicted mental health problems, *β* = .35, *p* < .001, thereby increasing the explained variance to 31%.

## Discussion

This study provides initial data on mental health differences between gay and bisexuals versus a population-based male sample in Germany. Furthermore, we noted that minority stressors significantly predict mental health problems in German gay and bisexual men above and beyond sociodemographic variables known to influence mental health such as age and education [28, 29].

Our investigation revealed that German gay and bisexual men report more mental health problems than a German male population sample. Our first hypothesis, namely that the gay and bisexual men would report more mental health issues than our population sample, was thus confirmed. These findings are consistent with those from other Western countries, including a number of meta-analytic data documenting mental health differences between gay and bisexual versus heterosexual men [1–3]. Furthermore, we detected a medium effect size in mental health differences that is in line with meta-analytic data [3]. Our study therefore provides additional evidence that mental health differences disfavoring gay and bisexual men seem to be a general issue in Western societies.

Furthermore, we found that younger participants and those with lower education levels reported slightly more mental health problems than older participants and those with higher education levels. These findings are consistent with previous research reporting better mental health in older individuals and those with higher socioeconomic status [28, 29].

In addition, we analyzed possible reasons for mental health problems within the gay and bisexual group: we found that victimization, rejection sensitivity, and internalized homonegativity significantly predict mental health problems in this sample. These findings are in line with previous studies documenting that minority stress predicts mental health problems in gay and bisexual men [11, 13, 16]. Hypothesis 2, stating that minority stress (victimization, rejection sensitivity, and internalized homonegativity) would predict mental health problems in gay and bisexual men, was thus confirmed as well.

In summary, we can assume that minority stress may be a driving factor in producing mental health differences between individuals afflicted by it, such as gay and bisexual men, and those who are not, such as heterosexual men. Accordingly, there is evidence that such mental health differences in German LGBs versus heterosexuals are mediated by sexual identity stress (stress based on the true sexual identity) [30].

Strengths of our study include the recruitment of a large sample of gay and bisexual men. Second, our results probably reflect a rather conservative estimate, since our sample of gay and bisexual men was compared to a population sample that might have included at least some gay and bisexual men who would have probably reported a higher mean of mental health problems than an entirely heterosexual sample.

Limitations of this study include that the gay and bisexual sample may not have been representative of German gay and bisexual men due to our online sampling method. However, since we controlled for sociodemographics in our analyses, sampling biases should be within reasonable limits. Second, we did not assess sexual orientation in the population sample and cannot therefore state how many gay and bisexual men it contained. Due to the low prevalence of a gay or bisexual orientation in men (about 2.8% in the U.S.) [23], we believe this limitation to be negligible. Third, our measure of mental health problems was rather weak and likely assessed psychological distress more than mental disorders.

A number of implications for clinical professionals can be drawn from this study: German gay and bisexual men are likely to suffer from more psychological distress and a greater number of mental health disorders than heterosexual men due to experiences of minority stress. They are therefore probably overrepresented in clinical samples. Psychotherapies for gay and bisexual men should thus take minority stress into account and find ways to diminish it or to improve the patients’ strategies to cope with it effectively. Other promising factors that may contribute to improving gay and bisexual men’s mental health are social support and feeling connected to the LGB community [16, 31–33]. Future studies should determine how diminishing minority stress and coping with it can be managed in order to reduce minority-stress linked psychological distress in gay and bisexual men.

## Conclusion

Our study indicates that mental health differences among German gay and bisexual men versus the general male population are prevalent. Research should focus on how to reduce and cope with minority stress. While a road to minority stress coping might be psychotherapy for inflicted individuals, minority stress reduction should also be pursued on a political level through mass media and general education about acceptance towards gay and bisexual orientations.

## Additional file

Additional file 1: Table S1. Used mental health subscales. (DOCX 31 kb)

## Abbreviations

ADHD: Attention-deficit/hyperactivity disorder
LGB: lesbians, gay men, and bisexual individuals
MHGGB: project “Minority Stress, Coping, Social Support, and Mental Health in German Gay and Bisexual Men” of the Philipps University Marburg
PTSD: Posttraumatic stress disorders
SCL-90-S: German Symptom-Checklist-90-Standard
U.S.: United States of America
VS: victimization scale
