# Supplementary material for: Mental health differences between German gay and bisexual men and population-based controls
Source: BMC Psychiatry. 2017 Jul 21;17:267. doi: 10.1186/s12888-017-1435-7 (PMC5521135; doi:10.1186/s12888-017-1435-7)
Supplement: Additional file 1: Table S1. — Used mental health subscales. (DOCX 31 kb) [file 12888_2017_1435_MOESM1_ESM.docx]

Additional file 1: Table S1

*Used Mental Health Subscales*

| Subscale | Item | Item Number |
| --- | --- | --- |
| Anger-hostility | Feeling easily annoyed or irritated | 11 |
|  | Temper outbursts that you could not control | 24 |
|  | Getting into frequent arguments | 74 |
| Anxiety | Feeling fearful | 33 |
|  | Spells of terror or panic | 72 |
|  | Feeling so restless you couldn’t sit still | 78 |
| Depression | Feeling lonely | 29 |
|  | Feeling no interest in things | 32 |
|  | Feelings of worthlessness | 79 |
| Paranoid ideation | Feeling others are to blame for most of your troubles | 8 |
|  | Feeling that that you are watched and talked about by others | 43 |
|  | Others not giving you proper credit for your achievements | 76 |
| Phobic anxiety | Feeling afraid in open spaces | 13 |
|  | Having to avoid certain things, places, or activities because they frighten you | 50 |
|  | Feeling uneasy in crowds | 70 |
| Psychoticism | The idea that someone else can control your thoughts | 7 |
|  | The idea that you should be punished for your sins | 85 |
|  | The idea that something is wrong with your mind | 90 |
| Somatization | Dizziness or nausea | 4+40 |
|  | Pains in heart or chest | 12 |
|  | Hot or cold spells | 49 |
| Interpersonal sensitivity | Feeling emotionally vulnerable | 34 |
|  | Feeling inferior to others | 41 |
|  | Strong inhibition when interacting with others | 69 |
| Obsessive-compulsive | Trouble remembering things and/or concentrating | 9+55 |
|  | Having to check and double-check what you do | 45 |
|  | Difficulty making decisions | 46 |

Note. The items “dizziness or nausea” and “having to check and double-check what you do” were adapted from the two items indicated in the gay and bisexual sample. In order to make the scores of the population sample comparable, in this sample the original items were averaged.
